# The effect of temperature exposure during shipment on a commercially available demineralized bone matrix putty

**DOI:** 10.1007/s10561-016-9578-1

**Published:** 2016-08-25

**Authors:** Mark Schallenberger, Helena Lovick, Jalane Locke, Todd Meyer, Gregory Juda

**Affiliations:** 1 DCI Donor Services Tissue Bank, 1714 Hayes Street, Nashville, TN 37203 USA; 2 Bacterin International, Inc., a wholly owned subsidiary of Xtant Medical Holdings, Inc., 664 Cruiser Lane, Belgrade, MT 59714 USA

**Keywords:** Shipping validation, Demineralized bone matrix, Allograft tissue, Osteoinductivity, Temperature effects

## Abstract

During August and September of 2013, temperature data loggers were shipped to and from an AATB accredited and FDA registered allograft tissue processing facility in Belgrade, MT (Bacterin International, Inc.) to five warm climate cities (Dallas, TX, El Paso, TX, New Orleans, LA, Phoenix, AZ, and Tampa, FL). Shipping data acquired from 72 independent shipments were analyzed to generate an assessment of temperature exposure, shipment times, and shipping event durations experienced during routine distribution. Overall the packages experienced an average temperature of 26.2 ± 2.3 °C which mirrored the average external ambient temperature of 25.8 ± 3.0 °C. However, temperature spikes above 40 °C were frequently observed. The data from the model shipments were extrapolated to provide a worst-case high temperature spike of 52.9 °C for 12 h and 14 min. Multiple lots of a commercially available demineralized bone matrix (DBM) putty (OsteoSelect^®^ DBM Putty) were subjected to continuous heating at 50 °C, to multiple worst-case temperature spikes, and to multiple freeze–thaw cycles to assess the effects of these temperature extremes on the handling and osteoinductivity of the allograft tissue. Five weeks of continuous exposure to 50 °C and 12 simulated worst-case one-way shipments did not adversely affect the handling characteristics or the in vivo osteoinductivity of the product.

## Introduction

The United States Centers for Disease Control and Prevention (CDC) estimates more than one million allografts are implanted annually in the United States (CDC Transplant Safety Overview 2015). Many of these allografts are stored and distributed at ambient temperature, including amniotic tissue, dermis, structural bone grafts, and demineralized bone matrix (DBM) products. Regardless of tissue type, all allografts are transported in some manner to the surgical facilities in which they are implanted. As it has been previously reported that packages can be exposed to extreme temperatures during routine shipment (Taylor 2001), it is incumbent on allograft processors to determine the effects of such transportation on the integrity of the allografts they distribute.

Because both low and high temperature extremes may be detrimental to allograft performance, federal regulations state that facilities must “establish appropriate shipping conditions to be maintained during transit” (21 CFR Part 1271 2004). Accordingly, the U.S. Food and Drug Administration (FDA) and the American Association of Tissue Banks (AATB) require manufacturers to establish acceptable temperature limits for storage of products and to perform on-going storage monitoring. While monitoring of these conditions is easily performed on-site, environmental monitoring during distribution is more challenging. Various solutions exist for temperature-controlled shipments (Ohkawara et al. 2012; Qin et al. 2013) but these options are not always physically or economically practical (Elliott and Halbert 2005; Singh et al. 2008).

Exposure to extremes in temperature has been shown to affect the mechanical and biological performance of a variety of human allograft tissues (Han et al. 2005; Shimp 2008). One commonly distributed form of allograft tissue particularly susceptible to thermal extremes is DBM (Russell and Block 1999; Urist 1965). The biologic activity, and by extension the clinical performance, of DBM is governed primarily by the protein growth factors associated with the bone collagen. The stability of bone protein growth factors has been reported to be negatively affected by prolonged exposure to temperatures above 45 °C. Additionally, the literature suggests that “wet” or hydrated DBM is more susceptible to thermal degradation than DBM in a lyophilized form (Shimp 2008). The heat stability of hydrated allografts throughout shipping warrants investigation since many commercially available DBM allografts are provided in a hydrated state due to the addition of carrier materials.

While previous studies have examined the effects of simulated transit conditions on protein formulations (Telikepalli et al. 2015), insulin samples (Chandler et al. 2008), essential drugs (Hogerzeil et al. 1992), and blood (Hankinson et al. 1989), a comprehensive evaluation of DBM stability during shipment has yet to be reported.

In this study, we sought to determine the temperature extremes allograft tissue may encounter during routine shipping conditions. We then tested the effects of such temperature exposure on both the physical characteristics and in vivo osteoinductivity of OsteoSelect^®^, a commercially available DBM putty. In order to assess the impact of elevated temperature on the DBM, a relevant industry adopted and FDA accepted in vivo cGMP lot release assay was used to evaluate the osteoinductive potential of the test article.

## Materials and methods

### Shipping conditions determination

In August and September of 2013, calibrated temperature loggers (SenseAware^SM^ 1000, FedEx and Hamster^®^-E EHT1, ELPRO) were shipped by air from Bacterin’s tissue processing facility in Belgrade, Montana to the warm climate cities of Dallas, TX, El Paso, TX, New Orleans, LA, Phoenix, AZ, and Tampa, FL. The temperature loggers were packaged and shipped in a manner representative of routine distribution of Bacterin’s allograft tissues. The packages containing the temperature loggers were received and then shipped back to the tissue processing facility in an identical manner as the outgoing shipments. The packages were shipped via a combination of overnight, 2-day, and weekend delivery (up to 4 days in transit) to account for a variety of shipping methods and potential delays in shorter-duration shipments. Multiple courier services were employed (FedEx^®^, UPS^®^, and USPS^®^) to account for potential differences between major shipping providers.

Once returned to Bacterin, the temperature data were compared to the reported ambient temperature for the city delivered to and against tracking information for the location of the package (inside jet, at sort facility, etc.). The data were analyzed to determine the impact of independent, external shipping factors on the internal package temperature. Specific shipping factors investigated included courier choice, duration of shipping events, and concurrent external temperatures.

**Fig. 1:**
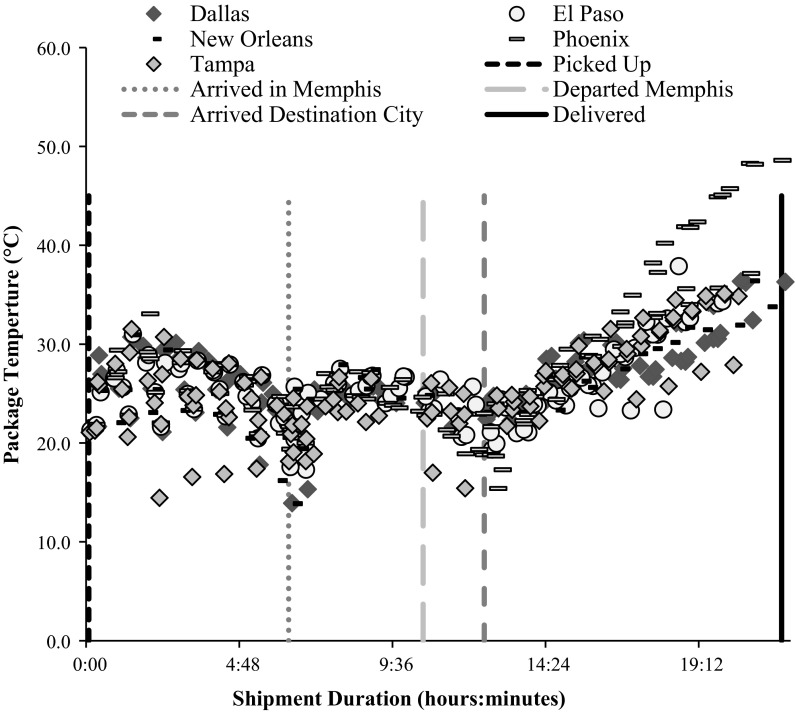
Temperature profile for 25 FedEx one-way, overnight shipments

**Fig. 2:**
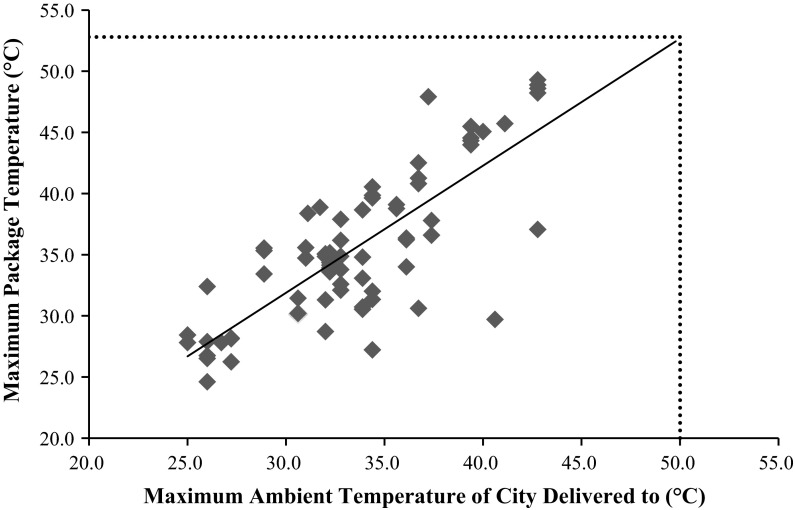
Maximum package temperature versus ambient temperature of destination city

Data from each shipment were compiled to generate a temperature exposure profile representative of routine distribution (Fig. 1). As the data demonstrated temperature spikes in excess of 40 °C, a plot was then generated to correlate average ambient temperature to these temperature spikes. For each individual shipment, the daily high temperature of the destination city was plotted against the maximum temperature the packages experienced during transit. Furthermore, to determine the worst-case temperature that could be expected during shipment, these data were extrapolated to include historical highs from the five cities (Fig. 2). This extrapolated temperature extreme was used for the subsequent allograft stability studies reported herein.

### Effects of worst-case shipping temperatures on a commercially available DBM putty

#### DBM putty

OsteoSelect^®^ DBM putty (Bacterin International, Inc.) was used for all testing detailed herein. OsteoSelect DBM Putty is a malleable bone grafting material comprising demineralized bone matrix (DBM) allograft (74 % by dry mass) suspended in a hydrated carboxymethyl cellulose (CMC) carrier (26 % by dry mass). The product is packaged in a polystyrene jar sealed within a high moisture barrier foil pouch and sterilized using a validated 9 kGy minimum gamma irradiation dose on dry ice to limit irradiation induced damage to the growth factors present within the graft. The product has been commercially available since 2009 and indicated for use as bone void filler and bone graft substitute in the extremities, pelvis, and posterolateral spine.

##### Prolonged heating

All allograft tissue utilized during the course of this study had confirmed research consent. The OsteoSelect DBM putty samples were exposed to continuous heating at 50 °C for 0, 1, 2, 3, 4, and 5 weeks in their final packaging to evaluate the effects of prolonged temperature elevation on allograft performance. The sample set comprised three different lots of OsteoSelect at each time point examined (a total of 54 grafts employed in this testing). For each time point, the osteoinductivity (OI) (n = 1 per lot) of the grafts was evaluated in a standard ectopic implantation assay in athymic rats (Edwards et al. 1998). This assay scores the histological evidence for new bone formation following 28 days of implantation and is a standard lot release assay for OsteoSelect DBM putty, (see additional detail below). The OI scores for the heated samples were compared to the OI scores of unheated, control samples using a paired, two-tailed *t* test. Additionally, for each time point the handling characteristics of the heated grafts (n = 2 per lot) were compared to non-heated control grafts from the same lot by three blinded evaluators to ensure the functional utility of the graft had not been altered as a result of the prolonged heating.

##### Temperature spike cycling

Following the completion of the prolonged heating experiments, additional DBM putty samples were exposed to temperature cycling to evaluate the effect of extreme temperature spikes, which may be encountered during routine distribution, on the performance of the allografts. To account for both hot and cold temperature spikes, DBM putty samples were exposed to either high-temperature cycles (52.9 °C for 12 h and 14 min, 23 °C for 12 h) or freeze–thaw cycles (−20 °C for 12 h, 23 °C for 12 h). The high-temperature and freeze–thaw cycles were selected to represent extreme temperature exposure for a one-way shipment. Multiple cycles were evaluated to account for the possibility of tissue returns and redistribution, which is common in the industry.

The testing comprised three different lots of recently processed DBM putty. Two samples from each lot were exposed to either: (a) no exposure (control samples), (b) seven high-temperature cycles, or (c) seven freeze–thaw cycles. For each condition examined, the osteoinductivity (OI) (n = 1 per lot) and handling characteristics (n = 1 per lot) of the grafts were evaluated in an identical manner as described for the continuous 50 °C heating sample set. A total of 18 grafts were employed for this testing.

Following the initial testing of recently processed DBM putty, follow-up testing was performed on three lots of 3-year old DBM putty to assess the effects of temperature exposure at the validated product family shelf-life (product stability data on file at Bacterin International, Inc.). Two samples from each lot were exposed to either: (a) no exposure (control samples), (b) twelve high-temperature spikes, or (c) twelve freeze–thaw cycles. Following the temperature exposure, OI testing (n = 1 per lot) and handling characterization (n = 1 per lot) were also performed. Again, a total of 18 grafts were employed for this testing.

The osteoinductivity (OI) scores were compared between groups a, b, and c for both sets of temperature cycling experiment samples using a paired, two-tailed *t* test. Table 1 provides a summary of the various temperature treatments and testing employed.

**Table 1:** Overview of DBM putty temperature treatments

Treatment name	Description	DBM putty details	Testing completed (per time point or per cycling group)
Prolonged heating	50 °C for 0–5 weeks	Recently processed Three different lots	n = 3 OI testing n = 6 handling evaluation
High-temperature cycling	One cycle = 52.9 °C for 12 h and 14 min, 23 °C for 12 h	Three lots of recently processed (7 total cycles) Three lots of 3 years old DBM putty (12 total cycles)	n = 3 OI testing n = 3 handling evaluation
Freeze–thaw cycling	One cycle = −20 °C for 12 h, 23 °C for 12 h	Three lots of recently processed (7 total cycles) Three lots of 3 years old DBM putty (12 total cycles)	n = 3 OI testing n = 3 handling evaluation

##### In vivo osteoinductivity (OI) testing in an athymic rat model

The OI testing employed an ectopic bone formation model in accordance with current industry-wide lot release testing standards. All OI testing was performed at WuXi AppTec (St. Paul, Minnesota, USA) using a validated, IACUC approved protocol. In brief, two implant sites per sample (250 ± 25 mg) were implanted into a surgically created intramuscular pouch of the *biceps femoris* muscles of athymic male rats. Each animal received two implants of DBM putty and samples were randomized so that no animal received the same lot in both non-bony implant sites. After 28 days, the animals were humanely euthanized and the implants were removed.

Tissues were fixed in 10 % neutral buffered formalin, decalcified, and processed into paraffin blocks. Sections were cut, slide mounted, and stained with hematoxylin and eosin (H&E stain). Final determination of osteoinduction was based on the semi-quantitative histopathology evaluation of multiple elements of new bone formation using an adapted version of the scoring system reported by Edwards (Table 2; Edwards et al. 1998). All histology was performed by an independent, board-certified veterinary pathologist. If elements of new bone formation are observed during histopathology, the test material is considered osteoinductive. If no elements of new bone formation are observed, the test materials are considered non-osteoinductive.

**Table 2:** Scoring of histologic sections (Edwards et al. 1998)

Score	New bone formation
0	No new bone
1	1–25 % of explant involved in new bone formation
2	26–50 % of explant involved in new bone formation
3	51–75 % of explant involved in new bone formation
4	>75 % of explant involved in new bone formation

## Results

### Worst-case temperature exposure during shipment

Eighty-eight packages were shipped as part of the testing to yield 72 useable one-way shipments and over 3300 h of shipment temperature data for analysis. Sixteen shipments were excluded due to excessive, non-representative shipping delays or problems with data acquisition by the temperature loggers. Details of the shipment method and destination cities for the 72 unique shipments are provided in Table 3.

**Table 3:** Overview of 72 unique one-way shipments to warm climate cities

Shipping method	Dallas	El Paso	New Orleans	Phoenix	Tampa	Totals
Overnight; outgoing^a^ to city	6	5	3	6	8	28
Overnight; returning^b^ from city	1	2	3	5	1	12
2-day; outgoing^a^ to city	2	3	4	4	3	16
2-day; returning^b^ from city	0	0	0	0	4	4
Weekend^c^	3	2	0	5	2	12
Totals	12	12	10	20	18	72

^a^All shipments outgoing to city originated from Belgrade, MT

^b^All shipments returning from city were sent to Belgrade, MT

^c^All weekend shipments were returning to Belgrade, MT from the destination, warm climate city. The weekend shipments included Thursday for Monday delivery, Friday for Monday delivery, and Friday for Tuesday delivery

Figure 1 provides a scatter plot of the temperature profile experienced during the 25 one-way overnight FedEx shipments to the five warm climate cities included in the testing. The average time points for shipping events are labeled on the chart. For a standard FedEx shipment, the five shipping events were (a) picked up at Bacterin, (b) arrival at Memphis, (c) departure from Memphis, (d) arrival at destination city, and (e) delivery (Note: Memphis is a FedEx shipping hub for all express packages). Average time elapsed (hours:minutes) at shipping events b–e, were 6:20, 10:33, 12:28, and 21:48, respectively.

As shown in Fig. 1, the temperatures of the interior of the packages were generally stable throughout shipment, with a trend towards increasing temperatures as the packages were on route for delivery in the destination city. A simple linear regression of the package temperature versus time in the destination city (shipping duration time points of 12:29–21:55) provided a coefficient of determination (R^2^) value of 0.68.

For the 72 unique, one-way shipments analyzed, the average internal temperature was 26.2 ± 2.3 °C mirroring the average external ambient temperature of 25.8 ± 3.0 °C. However, the packages routinely experienced spikes in temperature above 40 °C (for an average of 48 min with a range of 0 min–12 h and 14 min). These temperature spikes invariably occurred when the packages were on a vehicle for delivery or being loaded onto an aircraft. The maximum package temperature observed was 49.3 °C that originated from a shipment to Phoenix on a day when the high temperature exceeded 43 °C.

The maximum package temperature was plotted against the reported maximum ambient temperature for all the combined 72 one-way shipments (Fig. 2). The temperatures correlated with a R^2^ value of 0.62 for the linear regression line. This plot was extrapolated to include the historical highest temperatures for any of the cities examined (50 °C in Phoenix on June 26, 1990). By doing so, it was determined that the worst-case high-temperature spike a package would ever reasonably be subjected to is 52.9 °C. In order to set an extreme temperature exposure for subsequent testing, this high temperature was combined with the observed maximal amount of time a package was exposed to over 40 °C (12 h and 14 min).

### Concurrent shipping conditions

**Fig. 3:**
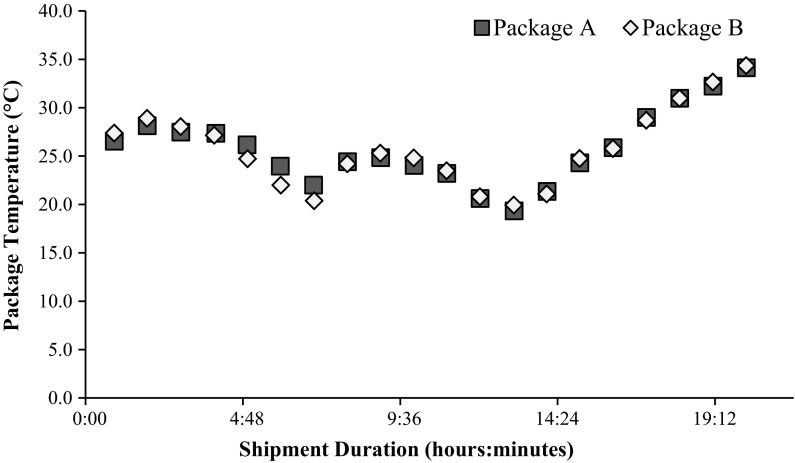
Temperature profile of two packages shipped concurrently from Bacterin to El Paso, TX

In order to test variability of temperature exposure of different packages shipped concurrently, 12 sets of paired packages were shipped as part of the 72 one-way shipments described above. These paired shipments were sent on the same shipping origination date, using the same shipment method (overnight, 2-day or weekend), and analyzed for differences once returned to Bacterin. All five warm climate cities were used as shipment destinations and originations in this sample set. For the majority of the paired shipments, the temperature profiles were nearly indistinguishable (average difference 0.6 ± 0.1 °C). Figure 3 provides a scatter plot representation of the temperature data collected for one such paired set of shipments mailed from Bacterin to El Paso, TX. The data demonstrate that the most important aspect of temperature exposure is the geographic location of the package (in flight, on route for delivery, etc.) and not its specific location within a transport vehicle.

### Courier services comparison

The temperature conditions encountered during four shipments to and from Tampa to Phoenix were also collected and analyzed for the three different courier services. All of these shipments originated on the same dates in September 2013. Table 4 provides a side-by-side comparison of the trip duration, maximum package temperatures and maximum ambient temperatures during the four, one-way shipments. The package temperatures during shipment were similar for all three couriers, with no statistically significant differences.

**Table 4:** Side-by-side comparison of four shipments by different couriers

Trip^a^	Data collected	Courier		
		FedEx	UPS	USPS
A	Trip duration (hours:minutes)	46:23	46:34	23:05^b^
B		93:40	89:42	96:43
C		20:20	15:21	19:32
D		50:06	51:42	47:06
A	Maximum ambient temperature (°C)	35.6	37.2	40.6
B		36.7	36.7	36.7
C		26.0	25.0	26.0
D		26.0	26.7	26.0
A	Maximum package temperature (°C)	38.8	47.9	29.7
B		40.8	42.5	30.6
C		27.9	27.8	24.6
D		32.4	27.8	26.5

^a^Trip A, 2-day shipment to Phoenix, AZ from Belgrade, MT; Trip B, Friday for Tuesday to Belgrade, MT from Phoenix, AZ; Trip C, Overnight shipment to Tampa, FL from Belgrade, MT; Trip D, 2-day shipment to Belgrade, MT from Tampa, FL

^b^The USPS shipment to Phoenix, AZ from Belgrade, MT arrived a day ahead of schedule

### Stability of a DBM putty under elevated temperature exposure

OsteoSelect DBM Putty grafts were exposed to continuous 50 °C heating as detailed in Table 5. Following temperature exposure, the handling characteristics of the grafts were evaluated by three blinded technicians and the osteoinductive potential of the grafts was evaluated using the standard in vivo lot release assay for the product family. The testing revealed that exposure to 50 °C had no effect on the handling characteristics of the grafts for all treatment groups (168–840 h). Additionally, all grafts maintained similar levels of osteoinductivity (no statistical differences) compared to control grafts from the same lots.

**Table 5:** Osteoinductivity results of DBM putty continuous 50 °C heating

Duration of temperature treatment	OI results^a^
1 week/168 h at 50 °C	100 % of controls (*p* = 1.00)
2 weeks/336 h at 50 °C	125 % of controls (*p* = 0.52)
3 weeks/504 h at 50 °C	100 % of controls (*p* = 1.00)
4 weeks/672 h at 50 °C	75 % of controls (*p* = 0.43)
5 weeks/840 h at 50 °C	75 % of controls (*p* = 0.43)

^a^Results from three separate lots

### Stability of a DBM putty exposed to worst-case shipping temperatures

**Fig. 4:**
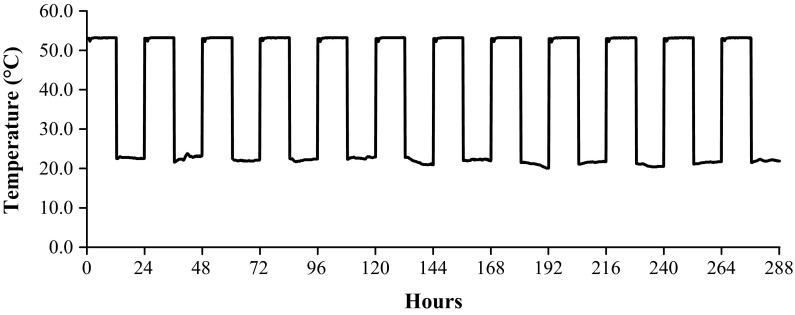
Temperature profile for 12 heat cycles of DBM putty

**Fig. 5:**
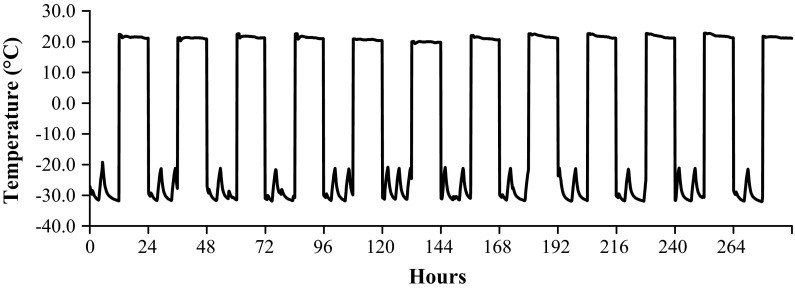
Temperature profile for 12 freeze–thaw cycles of DBM putty

To evaluate the effect of the worst-case shipping conditions identified above on the DBM, recently processed grafts were exposed to seven high-temperature cycles and seven freeze–thaw cycles. Furthermore, to ensure the potential temperature exposure did not affect the established shelf life of the product, 3 year old grafts were exposed to twelve high-temperature cycles and twelve freeze–thaw cycles. The testing details and results are reported in Table 6. Representative plots of the temperature profile for twelve high-temperature cycles and twelve-free–thaw cycles are provided in Figs. 4 and 5, respectively. None of the temperature treatments detectably affected the handling characteristics for any of the lots examined. All grafts received passing OI test results defined by histological evidence of new bone formation in the ectopic implant sites. The temperature exposure did not statistically affect the OI scores for any of the conditions or lots examined (all *p* > 0.05).

**Table 6:** Results of DBM putty temperature cycling

Temperature treatment	OI results
*Recently processed DBM putty lots* ^*a*^	
7 Heat cycles	100 % of controls (*p* = 1.00)
7 Freeze–thaw cycles	117 % of controls (*p* = 0.43)
*3* *year old DBM putty lots* ^*a*^	
12 Heat cycles	80 % of controls (*p* = 0.66)
12 Freeze–thaw cycles	80 % of controls (*p* = 0.66)

^a^Results from three separate lots

**Fig. 6:**
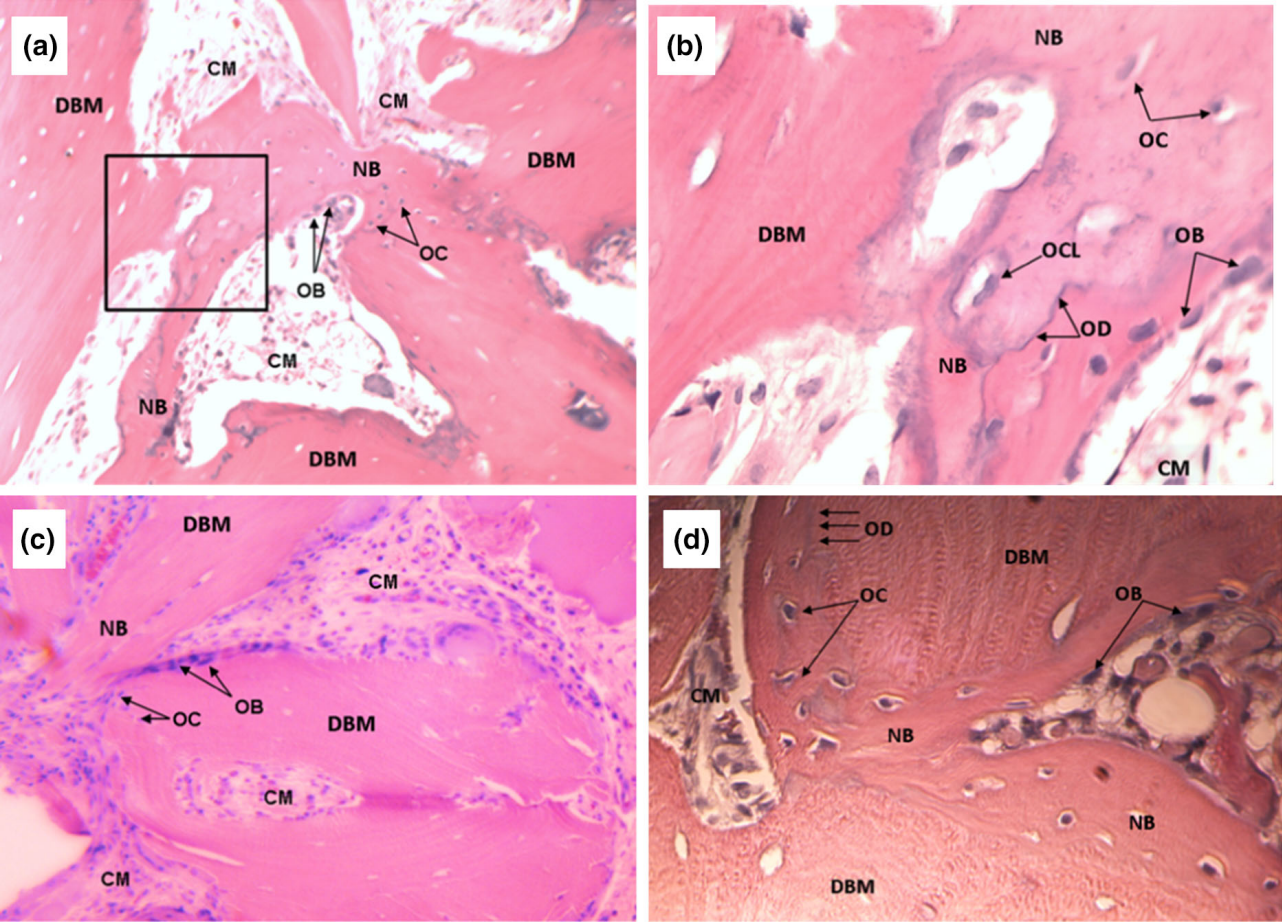
In vivo bone formation at 28 days post-implantation of OsteoSelect DBM putty in an athymic rat (H&E stain), three implant sites **a** ×50 magnification of implant site #1. **b** ×200 magnification of the *black box* region of implant site #1 shown in Fig. 6a. **c** ×50 magnification of implant site #2. **d ×**200 magnification of implant site #3. (*CM* condensed mesenchyme, *DBM* residual DBM implant, *NB* new mature bone, *OB* osteoblasts, *OC* osteocytes, *OCL* osteoclast, *OD* osteoid)

Four representative histology images of the in vivo bone formation at 28 days post-implantation of the temperature cycled DBM putty for three separate implant sites are provided in Fig. 6. The DBM putty implants are pictured rimmed with cells and new bone formation. The images shown in Fig. 6a, b are of the same implant site and differing magnifications. As shown in the enlarged image of Fig. 6b, there is a seamless transition between the residual implant material and newly formed bone. The remaining images, Fig. 6c, d illustrate new bone formation at two different implant sites.

## Discussion

Throughout the world, DBM allografts are widely employed in orthopedic surgery as bone graft substitutes and extenders (Drosos et al. 2015; Gruskin et al. 2012; Tilkeridis et al. 2014). While stability studies are routinely performed to ensure the grafts maintain their functional performance over the shelf-life of the product, the impact of extreme environmental exposure during distribution has received far less attention. The data presented herein demonstrate that even under express shipping conditions, allograft tissue may be exposed to temperatures in excess of 50 °C. As this is outside of the recommended storage conditions for most allograft tissues, it is of the upmost importance to test the effect of this potential temperature exposure on the biological performance of DBM allografts. This is especially important for allografts distributed in a hydrated state where free water can catalyze degradation of the proteinaceous growth factors present within the grafts (Shimp 2008). In this study, a thorough shipping validation was carried out on OsteoSelect DBM putty to ensure consistency of the product’s osteoinductive potential regardless of distribution conditions.

The osteoinductive potential of DBM upon implantation into a heterotopic site of a living animal was first described by Urist (1965). Later, a semi-quantitative measure was suggested by Edwards in order to provide a reproducible test method for the evaluation of the osteoinductive potential of DBM in a rat model (Edwards et al. 1998). Since then, variations of this in vivo assay have been widely adopted in the tissue banking industry for research evaluation, cGMP lot release testing, product stability testing, and regulatory submission of DBM containing medical devices featuring a claim of osteoinductive potential. In 2013, ASTM test method F2529 was published in an attempt to further standardize this basic test of osteoinductive potential, thus highlighting the importance of the in vivo assay (ASTM F2529-13). However, at the time of preparation of this manuscript, the ASTM test method has not been largely adopted across the allograft industry as a means of routine lot testing of 510(k) cleared DBM based products.

Out of over 3300 shipment hours, the study demonstrated that package temperatures were most impacted by the package’s geographic location and not significantly affected by the courier service type or the package’s specific location within the transport vehicle during distribution. In general, the average package internal temperature closely mirrored the average external ambient temperature, and it was noted that the maximum package temperatures had a linear correlation with, and could be predicted from, maximum ambient temperatures (Fig. 2). Additionally, temperature spikes above 40 °C were routinely observed. Based on historical temperature data, we extrapolated the worst-case high-temperature spike expected to be encountered during routine distribution in summer months (52.9 °C for 12 h and 14 min).

Following the evaluation of temperature extremes, OsteoSelect DBM putty was exposed to these worst-case shipping conditions to ensure the exposure did not adversely affect the biological activity of the product. The data collected demonstrated that at least 5 weeks of continuous 50 °C heat and 12 simulated worst-case one-way shipments did not adversely affect the osteoinductive potential or handling characteristics of the grafts.

Considering the robustness of the experimental protocol, it is somewhat unanticipated that the OsteoSelect DBM putty grafts maintained their biological activity throughout all conditions examined. It is known in the field that bone protein growth factors undergo degradation and inactivation upon high temperature exposure (Ijiri et al. 1994). Therefore, it is instructive to compare these results to those previously reported in the literature. In a landmark study published in 2005, Han and colleagues evaluated various heat exposures of DBM powder on its in vitro and in vivo osteoinductivity. Interestingly, the paper reported that while anhydrous DBM powder was largely unaffected at temperature exposure up to 55 °C for 5 weeks, DBM powder reconstituted in PBS lost an appreciable amount of OI when exposed to 45 °C for 5 weeks and a large amount of OI when exposed to 55 °C for 5 weeks (Han et al. 2005).

One possible explanation for the contrast between these results and those presented herein is that the CMC carrier of the OsteoSelect DBM Putty may function as a thermal protectant to the collagen and growth factors that compose DBM. Alternatively, the hygroscopic CMC carrier may serve to limit the amount of free water available to degrade the growth factors present in the DBM. Moreover, the intramuscular OI assay is likely insufficiently sensitive to detect minor reductions in osteoinductive potential. While it is possible that some degree of osteoinductivity was lost under the experimental conditions, we were unable to discern any statistically significant decrease within the sensitivity limits of the assay.

Although it is anticipated that an in vitro assay such as ELISA may provide a more quantitative measure of the thermal degradation of select protein growth factors within the DBM, these assays only measure one or a limited number of the biochemical markers associated with the induction of bone formation. Thus, the in vitro assays are only indirect measures of the osteoinductive potential of a DBM. In this study, we sought to utilize the most relevant assay model for the assessment of a DBM to induce bone formation. Although the in vivo model is not as quantitative as the in vitro assays, the in vivo evaluation is still appropriate given its widespread use in industry as the absolute measure of osteoinductive potential.

While we predict that further heating at temperatures above 50 °C will eventually inactivate the collagen associated growth factors, such excessively prolonged heating conditions would not be representative of real-world shipping conditions. Under the challenging, simulated shipping conditions employed in this study, this DBM putty maintained its initial handling characteristics and in vivo osteoinductivity. It is not known how generalizable these findings are to other DBM products with aqueous-based carriers. Therefore, we recommend allograft manufacturers perform a similar analysis for their products to ensure the integrity of their grafts is not compromised by temperature extremes during routine distribution.
